# Diabetes Causes Significant Alterations in Pulmonary Glucose Transporter Expression

**DOI:** 10.3390/metabo14050267

**Published:** 2024-05-07

**Authors:** Allison Campolo, Zahra Maria, Véronique A. Lacombe

**Affiliations:** Department of Physiological Sciences, College of Veterinary Medicine, Oklahoma State University, Stillwater, OK 74078, USA; allison.campolo@okstate.edu (A.C.); mariaz@okstate.edu (Z.M.)

**Keywords:** type 1 diabetes, lungs, GLUT trafficking, GLUT isoform, insulin

## Abstract

Diabetes has been identified as a significant and independent risk factor for the development or increased severity of respiratory infections. However, the role of glucose transport in the healthy and diseased lung has received little attention. Specifically, the protein expression of the predominant glucose transporter (GLUT) isoforms in the adult lung remains largely to be characterized in both healthy and diabetic states. Type 1 diabetes was induced via streptozotocin and rescued via subcutaneous semi-osmotic insulin pump for 8 weeks. The gene and/or protein expression of the most predominant GLUT isoforms from Classes I and III, including the major insulin-sensitive isoform (i.e., GLUT4) and novel isoforms (i.e., GLUT-8 and GLUT-12), was quantified in the lung of healthy and diabetic mice via qRT-PCR and/or Western blotting. Pulmonary cell surface GLUT protein was measured using a biotinylated photolabeling assay, as a means to evaluate GLUT trafficking. Diabetic mice demonstrated significant alterations of total pulmonary GLUT protein expression, which were isoform- and location-dependent. Long-term insulin treatment rescued the majority of GLUT protein expression alterations in the lung during diabetes, as well as GLUT-4 and -8 trafficking to the pulmonary cell surface. These alterations in glucose homeostasis during diabetes may contribute to an increased severity of pulmonary infection during diabetes and may point to novel metabolic therapeutic strategies for diabetic patients with concurrent respiratory infections.

## 1. Introduction

While diabetes is a relatively recent epidemic, respiratory infections like influenza, Severe Acute Respiratory Syndrome Coronavirus 2 (SARS-CoV-2), and pneumonia have plagued humans for millennia. However, it has only recently been determined that diabetic patients are three times more likely to die from respiratory infection as their non-diabetic counterparts [1,2], and that diabetic patients in particular are considered a high-risk group and should get vaccinated for flu [3,4]. In order to address this risk factor, therapeutic targets must be identified to assist diabetic patients with concurrent respiratory infection. However, very little is known about the regulation of glucose homeostasis in the lung.

It is thought that glucose diffusion into the airway surface liquid is mediated by epithelial permeability to glucose and the trans-epithelial glucose gradient, whereas glucose removal is regulated by facilitated cellular glucose uptake via the glucose transporters (GLUTs) [5,6]. These isoforms are categorized into three classes based on sequence similarity [7]. The Class I glucose transporters (GLUTs 1, 2, 3, and 4) are the most prominent and ubiquitously expressed GLUTs, whereas the Class III GLUTs (GLUTs 6, 8, 10, and 12) are the most novel isoforms and remain to be fully investigated. In addition, which GLUT isoforms are present and active in the adult whole lung of either humans or rodents has not been fully elucidated. Furthermore, how the regulation of pulmonary glucose transport might be altered during the diabetic state remains to be characterized.

Recent investigations in vitro have identified the mRNA of GLUTs 1–12 and both sodium-glucose-linked transporters (SGLTs) in human bronchial epithelial cells, but have only confirmed protein of GLUT1 and GLUT10 in these same cells [5]. To our knowledge, non-cancerous human whole lung tissues have been successfully probed only for SGLT1 [8] and GLUT4 protein [9]. Similarly, non-cancerous adult rodent lung tissue has only been successfully probed for the protein of SGLT1 and SGLT2 [5]. Clearly, there is a significant amount of inquiry into the functional GLUT proteins present in the adult mammalian lung. 

We hypothesized that GLUT-1, -2, and -10 protein expression will be predominant in the lung [5,10], which will be altered during diabetes. Thus, we here investigated the protein expression of the most predominant GLUTs from Class I (GLUT-1, -2, -3, -4) and Class III (GLUT-8, -10, and -12), which are important in mammalian glucose homeostasis in the lung of healthy and type 1 diabetic mice. Further, while some have previously speculated on the direct effect of insulin stimulation on the lung [11], to our knowledge, it remains unknown whether metabolic therapy such as insulin, one of the most used hypoglycemic agents, will directly affect the protein expression of pulmonary GLUT isoforms. Therefore, we here also explored how this therapeutic strategy for diabetic patients affects both GLUT protein expression and trafficking in the lung. 

## 2. Materials and Methods

### 2.1. Type 1 Diabetic Animal Model

The following experimental protocols were approved by the Oklahoma State University Institutional Animal Care and Use Committee, protocol #VM-18-3. Healthy FVB/N mice (The Jackson Laboratory, Ellsworth, ME, USA) received 3 intraperitoneal injections once every 48 h of either 300 mM citrate buffer or streptozotocin (Sigma Aldrich, St. Louis, MO, USA) dissolved in citrate buffer (at consecutive doses of 95 mg/kg, 65 mg/kg, and 95 mg/kg) to produce a type 1 diabetic mouse model. Once hyperglycemia was confirmed in type 1 diabetic mice (>250 mg/dL blood glucose), a subset of diabetic mice was given a subcutaneous semi-osmotic insulin pump (Alzet, Cupertino, CA, USA) at a dose of 0.5 units of HumulinRU-500 insulin (Eli Lilly, Indianapolis, IN, USA) per mouse per day. After 4 weeks, the insulin pumps were replaced. Mice were monitored weekly for blood glucose and weight for a total of 8 weeks. A total of 24 male mice were used in this animal model. At 8 weeks following the induction of diabetes, mice were humanely euthanized and lung (either whole lung or upper lung (primary and secondary bronchi) and lower lung (bronchioles and tertiary bronchi, separately) was collected and frozen until subsequent analysis. 

### 2.2. Real-Time Quantitative PCR (qRT-PCR)

Frozen samples (50–80 mg) were pulverized using Trizol reagent (Invitrogen, Waltham, MA, USA) according to the manufacturer’s instructions and RNA quantified using Gen5 software (v 2.22) with Biotek synergy HT hardware on a take3 plate (BioTek, Winooski, VT, USA). DNAse-treated RNA (Ambion AM1906M, Carlsbad, CA, USA) was then converted to complementary DNA using the High Capacity cDNA Reverse Transcription Kit and random primers (Applied Biosystems, Waltham, MA, USA) according to manufacturer’s recommendations and stored at −80 °C until analysis. The qRT-PCR assays for relative quantification of murine GLUT-1, -2, -3, -4, -8, -10, -12, and beta actin (Table 1) were performed using SYBR Green Real Time PCR Master Mix containing AmpliTaq Gold DNA Polymerase, to minimize nonspecific product formation, and deoxyribose nucleotide triphosphates with deoxyribose uridine triphosphate, to reduce carryover contamination (Applied Biosystems). Primer sequences were identified using the National Center for Biotechnology Information Basic Local Alignment Search Tool and custom synthesized by Invitrogen. Each PCR reaction (20 μL) contained 2X reaction buffer (SYBR Green I dye, Amplitaq Gold DNA Polymerase, deoxyribose nucleotide triphosphates with deoxyribose uridine triphosphate, passive reference, and optimized buffer components), forward and reverse primers (0.5 mM), 0.5 μg of complementary DNA, and DNase-RNase-free water. Primer concentrations and PCR conditions were determined during initial optimization runs. Samples were run in duplicate in a 96-well MicroAmp optical plate (Invitrogen). qRT-PCR was performed in an ABI 7500 Fast instrument (Applied Biosystems) with the following cycling conditions: 10 min at 95 °C, followed by 40 cycles at 95 °C for 15 s and 60 °C for 1 min. No-template, negative controls were included for each gene. A melting curve was generated to ensure product purity and the absence of primer dimers. The messenger RNA (mRNA) expression of target genes was normalized to β-actin, and the average cycle threshold was calculated.

### 2.3. Immunofluorescence Staining to Visualize GLUT4 in the Lung

In mice which did not have BAL fluid collected, the left lobe was perfused with paraformaldehyde and stored in formalin for 48 h or less before sectioning. Tissues were fixed with paraffin wax and sectioned in 4 µm slices onto slides, then stored in −20 °C until use. Upon rehydration and deparaffinization, slides were incubated at room temperature for three sets of 5 min in xylene, two sets of 10 min in 100% ethanol, two sets of 10 min in 95% ethanol, and two sets of 5 min in distilled deionized water. Slides were then washed in 1X TBST (Tris buffered saline + Tween 20) for five minutes before one hour of blocking (5% goat serum in TBST) at room temperature. Slides were then incubated overnight in primary antibody at 4 °C (GLUT4: Novus Biologicals, #NBP1-49533, Centennial, CO, USA). The next day, slides were allowed to incubate in primary antibody for 30 min at room temperature before incubation for thirty minutes with secondary antibody (GE Healthcare, #NA934, Chicago, IL, USA) at room temperature. Following the secondary antibody incubation, slides were washed with TBST wash buffer for three sets of 5 min, and cover slips were mounted using fluoroshield mounting medium with DAPI (AbCam, #ab104139, Cambridge, UK). Immunofluorescent slides were imaged the same day.

### 2.4. Photolabeling Biotinylation Assay

In order to assess GLUT trafficking to the cell surface, we quantified the protein expression of glucose transporters at the cell surface using the photolabeling biotinylation assay. To this end, lung tissue was finely minced and bathed in a Krebs–Henseleit buffer at 37 °C for 30 min, as described [12]. For insulin-treated lung homogenates, this buffer also contained 0.7 nm insulin. Both untreated (basal) and insulin-treated lung homogenates were then photolabeled with the cell surface impermeant biotinylated bis-glucose photolabeling reagent (bio-LC-ATB-BGPA, 300 µM, Toronto Research Chemicals, product #B3956740, North York, ON, Canada), of which the hexose group specifically interacts with the extracellular binding site of the GLUTs. This photolabeled reagent cross-linked to the lung homogenates using a Rayonet photochemical reactor (340 nm, Southern New England UV), as previously described [13]. Protein extraction was achieved via homogenization and ultracentrifugation (227,000× *g*, 90 min at 4 °C) to collect the crude membrane protein extract specifically. Recovery of photolabeled (cell surface, or “labeled”) GLUTs from total lung homogenates was achieved using streptavidin isolation (bound to 6% agarose beads, Thermo Fisher, product #20349, Waltham, MA, USA) to separate intracellular GLUTs (“unlabeled”) from cell surface GLUTs (“labeled”). The labeled GLUTs were then dissociated from the streptavidin via boiling in Laemmli buffer (Bio-Rad Laboratories, Hercules, CA, USA) for 30 min prior to Western blotting. Proteins from the unlabeled and labeled fraction were quantified by densitometry relative to the positive control, as previously described [12,13,14,15,16,17,18].

### 2.5. Western Blotting

Crude membrane protein extracts or total lysates were used for Western blotting. For total lysate creation, lung tissue was collected and homogenized in tissue lysis buffer consisting of a 1:500 dilution of RIPA (Thermo Fisher, product #89901, Waltham, MA, USA) and protease inhibitor cocktail (Sigma Aldrich, product #P8340, St. Louis, MO, USA). Protein content was quantified via bicinchoninic assay (Thermo Fisher, product #23227, Waltham, MA, USA), as previously described [13]. 

Equal amounts of protein (30 µg) were resolved in an 8–12% SDS-polyacrylamide gel and electrophoretically transferred (BioRad, Fort Worth, TX, USA) to a polyvinyl-idine fluoride membrane (BioRad), as previously described [13]. After blocking in 5% milk (BioRad), membranes were incubated in primary antibodies overnight on a rocker at 4 °C. Primary antibodies were diluted in tween-phosphate buffered saline (TPBS) with 5% milk (polyclonal rabbit anti-mouse GLUT1, 1:500 Abcam product #ab652; polyclonal rabbit anti-mouse GLUT2, 1:1000 polyclonal goat anti-mouse GLUT3, 1:500 Santa Cruz product #sc-7582; Abcam product #ab54460; polyclonal rabbit anti-human GLUT4, 1:750 AbD Serotec, product #4670-1704; polyclonal rabbit anti-human GLUT8, 1:500 Bioss product #bs-4241R; polyclonal rabbit anti-mouse GLUT10, 1:750 Thermo Fisher product #PA1-46137, polyclonal rabbit anti-mouse GLUT12, 1:500 Abcam product #ab75441; mouse monoclonal β-actin, Santa Cruz product #sc-47778). This was followed by a one-hour incubation at room temperature with an appropriate secondary antibody conjugated to horseradish peroxidase diluted in TPBS with 5% milk (for GLUT-1, -2, -10 and -12, 1:2500 donkey anti-rabbit IgG H&L Abcam product # ab7083; for GLUT3, 1:4000 Santa Cruz product #sc-2020; for GLUT-4 and -8, 1:3000 donkey anti-rabbit IgG HRP-linked GE Healthcare product #NA934; for β-actin, 1:5000 mouse IgG kappa binding protein Santa Cruz product #sc-516102). Primary antibodies were chosen based on their 100% sequence homology with the protein of interest in rodents, and validated against a positive control (GLUT1: liver or colon, GLUT2: kidney, GLUT3: testes, GLUT4: heart, GLUT8: heart, GLUT 10: liver, GLUT12: kidney), as previously described [12,13,14,16,18]. Antibody-bound transporter proteins were quantified by enhanced chemiluminescence reaction (Super Signal Max Sensitivity Substrate, product #34095, Thermo Fisher, Waltham, MA, USA) and autoradiography. Band density and molecular weight were quantified using GelPro Analyzer (Media Cybernetics, Rockville, MD, USA). Equal protein loading was confirmed by stripping (Thermo Fisher, product #21063, Waltham, MA, USA) and reprobing each membrane with β-Actin. The data were expressed relative to appropriate controls (i.e., positive and loading controls).

### 2.6. Statistical Analyses

Distribution and differences between means were assessed using a statistical software package (Sigmaplot 11.0, Systat Software, Inc., San Jose, CA, USA). Upon determining that datasets were normally distributed, using Shapiro–Wilk’s test, a two-way repeated measure ANOVA (treatment and time factors) for the in vivo measurements and a one-way analysis of variance (treatment factors) for the in vitro measurements were performed, as appropriate. When a significant difference was identified, post hoc tests were performed using the Student–Newman–Keuls test. Statistical significance was defined as *p* < 0.05. Data are presented as mean ± SE.

## 3. Results

### 3.1. Pulmonary GLUT Expression and Localization in Healthy Mice

**Figure 1:**
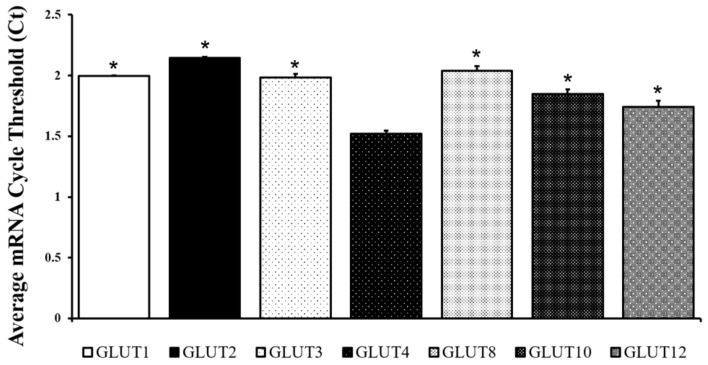
GLUT4 mRNA expression was the highest expressed GLUT isoform in the adult healthy mouse lung among those investigated. Mean ± SE of the average cycle threshold (Ct) of GLUT-1, -2, -3, -4, -8, -10, and -12. All values were normalized to beta actin, a housekeeping gene. A lower Ct value indicates a higher amount of mRNA. n = 4/group. * *p* < 0.05 vs. GLUT4 mRNA expression via two-tailed *t*-test.

In order to quantify the mRNA expression of predominant GLUT isoforms, healthy adult mouse lungs were collected, homogenized, and RNA was extracted using the Trizol method to conduct Real-Time Quantitative PCR (qRT-PCR). The average cycle threshold (Ct) value of GLUT-1, -2, -3, -4, -8, -10, and -12 mRNA was normalized to beta actin (Figure 1). A lower Ct value indicates a higher expression of mRNA. In the healthy adult mouse lung, GLUT4 was found to be the highest expressed GLUT isoform, followed by GLUT12 (*p* = 0.008), then GLUT10. While the proteins of GLUT-1, -2, -4, -8, -10, and -12 were confirmed subsequently through Western blotting (Figures 4 and 5), GLUT3 protein was not found to be present in the adult mouse lung via Western blot. 

To determine where GLUT4 may be localized in the healthy adult mouse lung, we employed immunofluorescent staining (Figure S1). This staining indicated that GLUT4 was expressed in both the upper lung (primary and secondary bronchi, Figure S1A) and the lower lung (bronchioles and tertiary bronchi, Figure S1B). 

To determine ex vivo insulin-stimulated GLUT trafficking in the lung, we first separated the upper lung and lower lung in order to estimate prevalence of GLUT protein in either the bronchial epithelial cells (upper lung, Figure S1C) or alveolar type I and type II cells (lower lung, Figure S1D). In the upper lung, lung homogenates from healthy mice were finely minced and incubated for 30 min with either just KHB buffer (basal) or KHB buffer plus insulin. Lung homogenates were then incubated with the biotinylation photolabeled compound, and cell surface GLUT protein was quantified via Western blot as a means to evaluate GLUT trafficking to the cell surface. The predominant insulin-sensitive glucose transporter, GLUT4, was only detected at the cell surface of the upper lung, and none whatsoever was detected at the cell surface of the lower lung. In the upper lung, there was no difference in GLUT4 cell surface protein expression between basal- and insulin-stimulated lung homogenates. 

### 3.2. Validation of the Diabetic Animal Model

**Figure 2:**
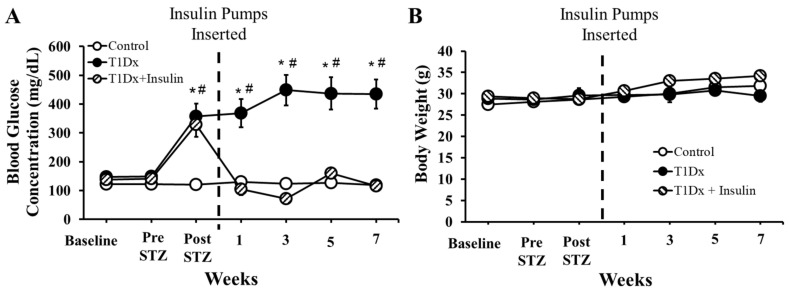
Insulin treatment rescued hyperglycemia, without affecting body weight, in type 1 diabetic animals. Mean ± SE of fasted serum blood glucose levels of (**A**) type 1 diabetic (T1Dx). T1Dx were treated with insulin (T1Dx + Ins) for 8 weeks. Mean ± SE of body weight of (**B**) type 1 diabetic (T1DX). n = 9–12/group. * *p* < 0.05 vs. control, # *p* < 0.05 vs. baseline, via two-way repeated measure ANOVA.

Healthy mice were injected with streptozotocin to induce type 1 diabetes (as indicated by pronounced hyperglycemia, greater >250 mg/dL) and a subset was subsequently treated with a subcutaneous insulin pump up to 8 weeks after the induction of diabetes (Figure 2A). After STZ injection, untreated diabetic mice maintained a blood glucose level significantly higher than control or treated counterparts (*p* < 0.01), and higher than themselves at baseline (*p* < 0.01). Insulin treatment restored euglycemia in diabetic mice within one week after the beginning of the treatment (Figure 2A). There were no significant differences in weight between groups in the type 1 diabetic study (Figure 2B). 

### 3.3. Pulmonary GLUT Protein Expression during Diabetes

**Figure 3:**
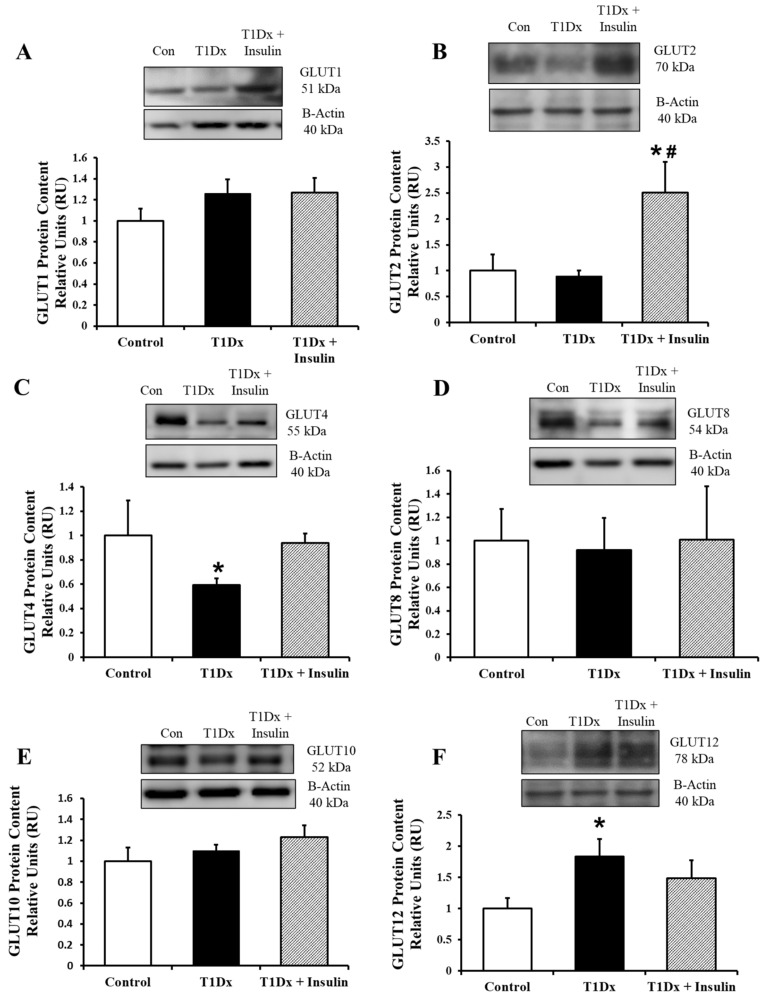
Alterations in glucose transporter (GLUT) protein expression in the adult upper lung during type 1 diabetes were rescued by in vivo insulin treatment. Total protein expression of (**A**) GLUT1, (**B**) GLUT2, (**C**) GLUT4, (**D**) GLUT8, (**E**) GLUT10, and (**F**) GLUT12 in the adult whole lung of control, type 1 diabetic (T1Dx), and insulin-treated animals (T1Dx + Insulin). Top panels: representative Western blot from total lysate; loading control: beta actin. Bottom panels: mean ± SE of total GLUT protein content (values normalized to beta actin and respective controls, n = 4/group). * *p* < 0.05 vs. control, # *p* < 0.05 vs. T1Dx, via one-way ANOVA. Methods: Western blotting.

**Figure 4:**
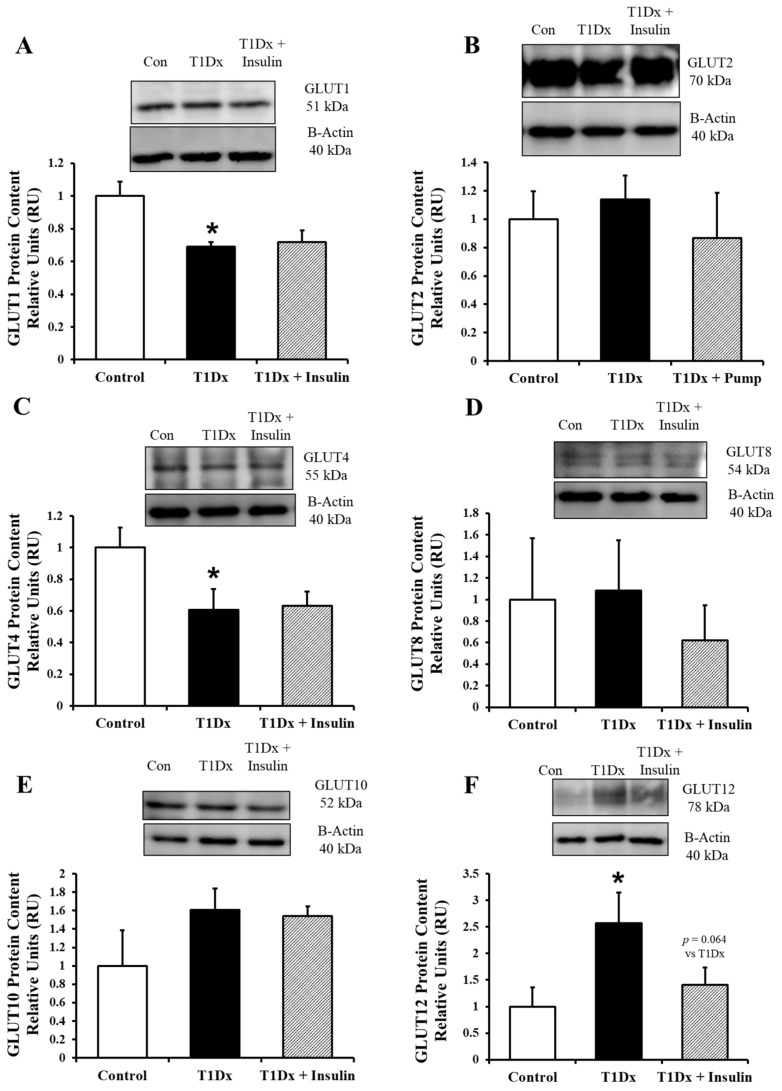
Alterations in glucose transporter (GLUT) protein expression in the adult lower lung during type 1 diabetes were rescued by in vivo insulin treatment. Total protein expression of (**A**) GLUT1, (**B**) GLUT2, (**C**) GLUT4, (**D**) GLUT8, (**E**) GLUT10, and (**F**) GLUT12 in the adult whole lung of control, type 1 diabetic (T1Dx), and insulin-treated animals (T1Dx + Insulin). Top panels: representative Western blot from total lysate; loading control: beta actin. Bottom panels: mean ± SE of total GLUT protein content (values normalized to beta actin and respective controls, n = 4/group). * *p* < 0.05 vs. control, via one-way ANOVA. Methods: Western blotting.

In order to determine the difference, generally, between the predominant cells of the upper lung (respiratory epithelial cells of the bronchi and bronchioles) and the lower lung (type 1 and type 2 alveolar cells), the upper and lower lung were separated at collection of the type 1 diabetic animals. The upper and lower mouse lung was homogenized and the protein was quantified via Western blot. In the upper lung total lysate, only GLUT4 protein expression was decreased, while GLUT12 protein expression was increased during type 1 diabetes (*p* = 0.038 and *p* = 0.033 vs. control, respectively, Figure 3C and Figure 4F). Insulin treatment fully rescued both of these alterations. Also, while there was no difference in GLUT2 protein expression between control and type 1 diabetic animals, the insulin-treated animals demonstrated a significant upregulation of GLUT2 content (*p* = 0.039 vs. control, Figure 3B). 

In the lower lung total lysate, GLUT1 and GLUT4 had a significantly lower amount of protein expression in type 1 diabetic animals versus their control counterparts (*p* = 0.014 and *p* = 0.048 vs. control, respectively, Figure 4A and Figure 4C, respectively). Similar to the upper lung total lysate, GLUT12 protein expression was observed in the lower lung of type 1 diabetic animals (*p* = 0.0438 vs. control, Figure 4F). All of these alterations were rescued with long-term insulin treatment. Finally, similar to the upper lung, no change in GLUT10 protein expression was observed in treated and untreated diabetic mice.

**Figure 5:**
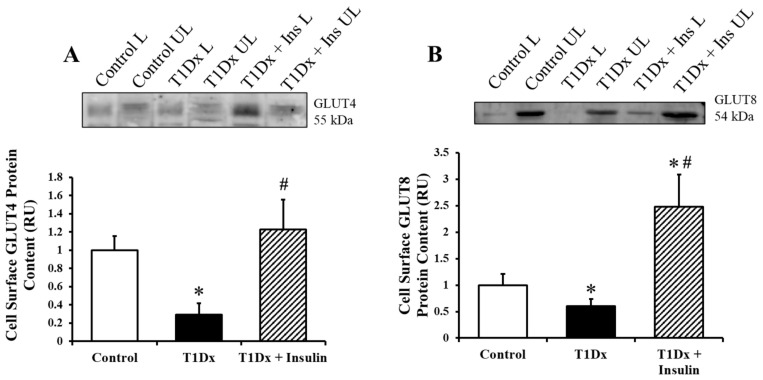
Alterations in pulmonary cell surface glucose transporter (GLUT) protein expression during type 1 diabetes were rescued by in vivo insulin treatment. Cell surface protein expression of (**A**) GLUT4, and (**B**) the novel GLUT8 isoform at the cell surface of the adult whole lung of control, type 1 diabetic (T1Dx), and insulin-treated animals (T1Dx + Insulin). Top panels: representative Western blot. Bottom panels: mean ± SE of cell surface GLUT protein content (values normalized to respective controls, n = 4/group). * *p* < 0.05 vs. control, # *p* < 0.05 vs. T1Dx, via one-way ANOVA. L: labeled (cell surface fraction); UL: unlabeled (intracellular fraction). Methods: photolabeling biotinylation assay.

To determine trafficking of insulin-sensitive GLUTs to the cell surface, whole lung homogenates of healthy, treated, and untreated diabetic mice were finely minced and incubated with the biotinylation photolabeled compound. Cell surface GLUT-4 and -8 protein expression, quantified via Western blotting, was significantly downregulated in the lung of type 1 diabetic mice compared to control counterparts (*p* = 0.001), which were rescued with long-term insulin treatment (*p* = 0.022 and 0.005, respectively, Figure 5). Similar findings were reported for the novel GLUT8 isoform.

## 4. Discussion

In mammals, the GLUT family consists of 14 different isoforms that differ in their tissue expression patterns, substrate specificity, and transport kinetics. Importantly, GLUT1, a ubiquitously expressed isoform, is a membrane-bound GLUT that facilitates the influx of glucose into the cell at a basal rate. Other GLUTs, such as GLUT4, require activation by insulin to translocate from an intracellular vesicle to the cell surface in order to enable glucose diffusion into insulin-sensitive tissues. To the best of our knowledge, the protein expression of only a few GLUT isoforms have been reported in non-cancerous adult whole mammal lung (including rodents and humans) [5,19], although recent data have indicated that GLUT 1 and GLUT4 protein are also altered in the lung during SARS-CoV-2 infection in felines [20,21]. This study comprehensively evaluated both the gene and protein expression of the most predominant GLUT isoforms of Classes I and III in the lung of adult healthy mice in the same study. We characterized the presence of several GLUT isoforms from Class I (GLUT-1, -2, -3, and -4), as well as from Class III, which includes GLUT10 as well as novel isoforms such as GLUT8 and GLUT12 in the adult lung of healthy mice. The data reported in this study suggest that GLUT4, the predominate insulin-sensitive GLUT isoform, and the novel transporters GLUT8 and GLUT12 are the most likely candidates to regulate respiratory glucose homeostasis. Further, there is growing evidence that the multiple types of cells within the lungs express different GLUTs and likely respond to alterations in glucose levels differently. Therefore, while other studies have used in vitro cell culture techniques to detect the different levels of GLUTs in different pulmonary cell types, we macroscopically identified differences in regional heterogeneity by separating the upper lung (predominately the bronchiolar cells) and the lower lung (predominately the alveolar cells) in adult mice. 

As the predominate insulin-sensitive GLUT in the body, we also sought to determine whether pulmonary GLUT4 trafficking could be stimulated ex vivo with acute insulin treatment (via 30 min insulin incubation of lung homogenates). Although GLUT4 has been detected in human trachea, bronchioles, and primary human bronchial epithelial cells [5,19], to our knowledge, no studies have described the specific insulin response as it relates to this major transporter in this tissue. The current study described the presence and activity of GLUT4 in adult mouse lungs (as opposed to in vitro studies or human samples) [9]. For instance, we performed our well-established biotinylation photolabeling assay that provides a direct and easily quantifiable measure of active cell surface GLUTs as a means to evaluate GLUT trafficking, the rate-limiting step in glucose uptake. We successfully quantified the cell surface GLUT protein content in the lung, including for the novel GLUT8 isoform. In addition, unlike reports in striated muscle and adipose tissue [12,13,16,17,18], we found no evidence that GLUT4 trafficking was directly stimulated by insulin in lung homogenates. Furthermore, we only found evidence of GLUT4 at the cell surface of the upper lung, and no GLUT4 protein at all in the cell surface of the lower lung using a macroscopic dissection technique combined with photolabeling technique. The former technique allows us to generally differentiate the two predominate morphologies of the lung (more bronchial epithelial cells in the upper lung, and more alveolar type 1 and type 2 cells in the lower lung). These results would indicate that GLUT4 at the cell surface is predominate in the bronchial epithelial cells compared to in the alveolar cells. 

It has recently become apparent that diabetic patients are at a significant risk for pulmonary infection (e.g., influenza, SARS-CoV-2) [1,2,3], and glucose homeostasis in the lung has so far remained elusive. Recent investigations have identified that there is a relatively constant ratio of glucose in the airway surface liquid to blood glucose (roughly 12.5 times as much glucose in the blood as the airway) [22] and that this glucose diffuses passively into the airway [5]. However, it is theorized that glucose removal from the airway is conducted primarily via GLUTs in the respiratory system [5]. Thus, understanding the specific protein presence and GLUT activity of the airway is of crucial importance to unravel the complications causing the increased risk of pulmonary infection in diabetic patients. 

In order to examine this hyperglycemic risk as exhaustively as possible, we here used a type 1 diabetic mouse model. Similarly, in order to prevent the introduction of variables from genetic variants, we did not use transgenic mouse models of diabetes. Instead, streptozotocin has long been a reliable method of inducing severe hyperglycemia [23], while maintaining normal body weight and insulin sensitivity. It is critically important to understand what alterations are due to hyperglycemia alone versus those due to hyperinsulinemia, inflammation, and obesity. In the present study, we investigated the alterations of glucose homeostasis of the lung as it relates to type 1 diabetes. We also chose to treat our diabetic mice with insulin in order to determine if diabetic alterations of pulmonary GLUTs were reversible, and if these common treatments already readily used in human patients were effective. As expected, insulin treatment fully rescued the pronounced hyperglycemia and the majority of the GLUT alterations found in the lung of type 1 diabetic mice. 

Following these in vivo conditions, we quantified the protein expression of GLUTs in the upper and lower lung. In the upper lung, GLUT4 was significantly downregulated in type 1 diabetic animals, while GLUT12 was significantly upregulated. Both of these changes were rescued with insulin treatment. And while no other GLUTs in the total lysate of the upper lung were altered in type 1 diabetic animals, we were surprised to find a significant upregulation of GLUT2 in the insulin-treated animals as compared to both the control and the untreated diabetic mice. This GLUT12 upregulation has been previously reported, including in the kidney, in conjunction with a downregulation of GLUT4, and is speculated to be part of a compensatory response [14]. In the lower lung of type 1 diabetic mice, both GLUT-1 and -4 were significantly downregulated as compared to controls. GLUT12 protein was significantly upregulated in type 1 diabetic mice. These alterations were completely rescued by insulin treatment, and there were no alterations of GLUT10 protein in treated and untreated diabetic mice. These data suggest that GLUT10 may not be sensitive to changes in insulin or glucose homeostasis, and that it functions as a basal GLUT only. In addition, GLUT protein expression was altered in a similar manner in both the upper and lower lung during hyperglycemia. Thus, the only differences in GLUT protein expression between the upper and lower lung appear to be GLUT1 and GLUT2. This would point toward regional heterogenicity in glucose transport (e.g., bronchial epithelial cells vs. alveolar type I or type II cells). In order to more fully investigate these regional differences, additional studies using immunohistochemistry, immunofluorescence, or cell isolation should be conducted.

We here additionally quantified the cell surface protein expression of GLUTs in the lungs of type 1 diabetic mice and compared those results with those from healthy and insulin-treated diabetic mice. Since GLUT-1, -2, -3, and -10 are glucose transporters which reside predominately on the cell surface and do not rely on trafficking mechanisms to function [24], we did not measure the specific expression of these GLUTs at the cell surface. GLUT12, a novel Class III GLUT, has also been characterized as a basal cell surface in striated muscle [14]. However, both GLUT4, the predominant insulin-sensitive glucose transporter, and GLUT8, another novel Class III glucose transporter, rely on trafficking from an intracellular pool to the cell surface after stimulation from either insulin or calcium (15). Thus, we measured the cell surface and intracellular fractions of these two insulin-sensitive GLUT isoforms as a means to estimate GLUT trafficking to the cell surface. We determined that while GLUT4 protein was decreased in both the lung total lysates and at the pulmonary cell surface, GLUT8 protein was only decreased at the cell surface during type 1 diabetes, but remained unchanged in both the upper and lower lung total lysates. This further indicates that GLUT8 may be an active participant in pulmonary glucose homeostasis and may rely on trafficking mechanisms in order to function in the airway. The alterations in cell surface GLUT-4 and -8 protein expression were rescued by insulin treatment. However, since acute insulin stimulation did not increase GLUT4 trafficking ex vivo in lung homogenate (which is consistent with our findings in vitro [25]), it is likely that this in vivo metabolic treatment rescued GLUT protein expression in the lung due to a rescue of whole-body glucose homeostasis rather than a specific pulmonary regulation. However, additional studies using greater sample size will be required to characterize the regulation of glucose transport in the lung to confirm the data demonstrated here and by others [9].

In conclusion, we have quantified the gene and protein expression of predominant GLUT isoforms in the adult lung of healthy, diabetic, and treated diabetic animals. Of the six isoforms for which we found protein in non-cancerous lung tissue (GLUT-1, -2, -4, -8, -10, and -12), all of the isoforms (with the exception of GLUT10) were determined to have some sort of alteration during diabetes, either in the total cell lysate or at the cell surface of the homogenized lung tissue. Long-term insulin treatment rescued the majority of the alterations in GLUT protein expression and/or trafficking during diabetes. Overall, the results from this study indicate that diabetes significantly alters GLUT protein expression in the adult lung, and targeting the maintenance of function of the pulmonary glucose transporters may designate a significant treatment avenue for diabetic patients with concurrent respiratory infections. Therefore, insights from this study could identify novel metabolic therapeutic strategies for diabetic patients with concurrent respiratory infections.

## Figures and Tables

**Table 1:** Sequence, accession numbers, and product size for qRT-PCR primers used.

Gene Name	Sequence (5′-3′)	Accession Number	Product Size
Beta Actin Forward	GAT TAC TGC TCT GGC TCC TAG	NM_007393.5	147
Beta Actin Reverse	GAC TCA TCG TAC TCC TGC TTG		
GLUT1 Forward	GGC TGA TTG GTG ACT TGT TGG	D10229.1	142
GLUT1 Reverse	GTG GAA CTG GTG AGT CTG GG		
GLUT2 Forward	CAG CTG TCC CTG TCC CAT TT	X78722.1	117
GLUT2 Reverse	AGC CTG ACC TGT GGT AAC TG		
GLUT3 Forward	GAC AGA CTA GGT GTG CCT GG	NM_011401.4	108
GLUT3 Reverse	GGT TTG TGA GAG GCC ATG TTT		
GLUT4 Forward	GTA ACT TC TTG TCG GCA TGG	NM_009204.2	128
GLUT4 Reverse	AGC TGA GAT CTG GTC AAA CG		
GLUT8 Forward	CTT CGT GAC TGG CTT TGC TG	NM_019488.5	102
GLUT8 Reverse	AAC ACC ATG ATC ACA CCC GA		
GLUT10 Forward	GGG CCT GAC CTT CGG ATA TG	NM_130451.3	140
GLUT10 Reverse	AGC GAA AGA TGG TAG AGG CG		
GLUT12 Forward	CCT GCC CTC AGG AAT CAC TC	NM_178934.4	108
GLUT12 Reverse	AGA CTG GGA CCA TTT GGT GG		

## Data Availability

Data will be made available upon reasonable request.

## Supplementary Materials

The following supporting information can be downloaded at: https://www.mdpi.com/article/10.3390/metabo14050267/s1, Figure S1. GLUT4 protein is present in the upper and lower lung, and pulmonary cell surface GLUT4 protein expression is not significantly altered after insulin treatment ex vivo. (A) Representative images from immunofluorescent GLUT 4-stained whole left lungs in bronchioles and (B) terminal and respiratory bronchioles and alveoli in healthy adult mice Blue: DAPI, Green: GLUT4. Methods: Immunofluorescence (C) Cell surface protein expression of GLUT4 in the upper lung, and (D) lower lung of lung homogenates treated with buffer (Basal) or with 0.7 nm insulin for 30 min. No cell surface protein expression of GLUT4 is evident in the lower lung, indicating only whole cell lysate lower lung GLUT4 protein expression. Top panels: representative Western blot. Bottom panels: Mean ± SE of cell surface GLUT protein content (values normalized to respective controls, n = 3–5/group). L: Labeled (cell surface fraction); UL: Unlabeled (intracellular fraction). Methods: Photolabeling biotinylation assay.
