# Supplementary figures and images for: Diabetes Causes Significant Alterations in Pulmonary Glucose Transporter Expression
Source: Metabolites. 2024 May 7;14(5):267. doi: 10.3390/metabo14050267 (PMC11123172; doi:10.3390/metabo14050267)

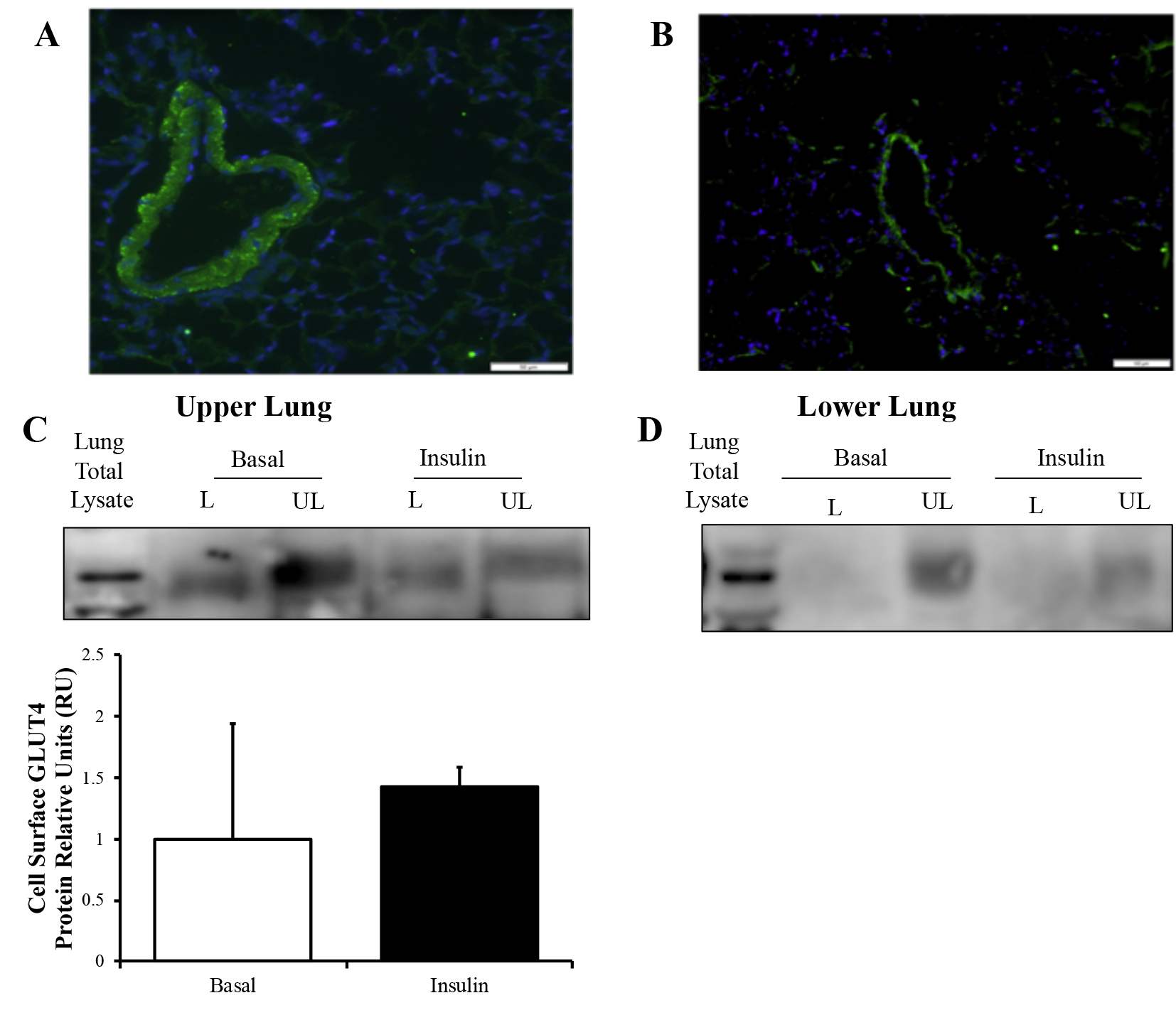

Supplement: Supplementary file 1 [file metabolites-14-00267-s001.zip › metabolites-2953954-supplementary.tif]
